# HIV 2-LTR experiment design optimization

**DOI:** 10.1371/journal.pone.0206700

**Published:** 2018-11-08

**Authors:** LaMont Cannon, Cesar A. Vargas-Garcia, Aditya Jagarapu, Michael J. Piovoso, Ryan Zurakowski

**Affiliations:** 1 Department of Biomedical Engineering, University of Delaware, Newark, DE, United States of America; 2 Department of Electrical and Computer Engineering, University of Delaware, Newark, DE, United States of America; 3 Department of Pathology & Laboratory Medicine, University of Pennsylvania, Philadelphia, PA, United States of America; 4 Fundación Universitaria Konrad Lorenz, Bogota, Colombia; George Mason University, UNITED STATES

## Abstract

Clinical trials are necessary in order to develop treatments for diseases; however, they can often be costly, time consuming, and demanding to the patients. This paper summarizes several common methods used for optimal design that can be used to address these issues. In addition, we introduce a novel method for optimizing experiment designs applied to HIV 2-LTR clinical trials. Our method employs Bayesian techniques to optimize the experiment outcome by maximizing the Expected Kullback-Leibler Divergence (EKLD) between the a priori knowledge of system parameters before the experiment and the a posteriori knowledge of the system parameters after the experiment. We show that our method is robust and performs equally well if not better than traditional optimal experiment design techniques.

## Introduction

Amid the increasing costs of carrying out experiments coupled with a decreasingly generous funding environment, there is an expanding charge to apply optimization methods to clinical trial design in order to maximize the amount of information that can be garnered from the resulting data [1–4]. This is especially true in the case of clinical trials used in biomedical research. Not only is the monetary cost a principal concern, when the study contains human subjects, the overall burden to the patient must be also considered. The latter is meticulously controlled under regulations imposed by the Institutional Review Board (IRB) [5].

With these considerations in mind, this paper focuses on methods which can be used to find the optimal design criteria that will maximize the information content in an experiment to gather knowledge about the in vivo dynamics of the Human Immunodeficiency Virus (HIV). Advances in combination antiretroviral therapy (cART) for treatment of HIV have drastically reduced Acquired Immune Deficiency Syndrome (AIDS) related mortality rates worldwide [6]. Clinical Analysis has shown that cART is able to suppress viral levels below the limit of detection; however, complete eradication is not achieved [6–12], most likely due to the presence of long-lived quiescent infected cells capable of re-seeding the HIV reservoir once treatment is discontinued [8–11, 13]. In addition to the long-lived reservoir formed by quiescently infected T-cells, evidence is mounting for the existence of ongoing replication of HIV within a subset of patients treated on cART [14]. This is a controversial topic in HIV research [3, 14–16]. Strong evidence for a lack of ongoing replication exists. Multiple phylogenetic studies have shown no longitudinal evolution of HIV in treated patients [17–20]. Intensification studies have been shown to have no measurable effect on the residual HIV RNA levels in the plasma [21–24]. Conversely, lymphoid biopsy studies have shown dramatically reduced levels of certain antiretroviral drugs in primary lymph nodes as well as gut-associated tissues; these decreased antiviral concentrations were associated with increased levels of HIV RNA consistent with localized HIV replication [25]. Also, one recent study has shown evidence of viral evolution in suppressed patients [26]. Finally, raltegravir intensification studies have shown transient increases in 2-LTR that are best explained as evidence of localized ongoing HIV replication prior to intensification [26–29]. Unfortunately, preliminary studies which aimed to test for the presence of biomarkers of replication yielded inconclusive results [28, 30, 31]. We believe that this was due to sub-optimal experiment designs, thus the aim of this paper is to explore several experiment design optimization methods that will allow for more informative results.

These studies have sought to detect on-going replication by intensifying antiretroviral therapy with an integrase inhibitor [28, 30, 31]. In the presence of an integrase inhibitor viral DNA is unable to integrate into the host genome. Host nuclear enzymes convert this un-integrated DNA into circles with two long term repeat ends. These converted DNA elements, which are aptly referred to as 2-LTR circles, serve as a marker of on-going replication [28, 30–32]. In vivo 2-LTR concentrations are estimated using the polymerase chain reaction (PCR) method on blood samples drawn from patients under integrase inhibitor intensification. If a high level of on-going replication is present, a transient increase in the 2-LTR concentration is expected [28, 30–32]. There will initially be a sharp increase in production of 2-LTR circles as the new infections are inhibited and 2-LTR circles are formed, but the production will then decrease since the success rate of infection drastically decreases [28, 30–33]. These 2-LTR circles are widely thought to be transcriptionally inert, but there is some evidence of low levels of transcriptional and translational activity from them in vitro, potentially even resulting in replication-competent integrated infections once integrase inhibitors are discontinued [34, 35]. While this is concerning with respect to a possible infection rebound subsequent to discontinuing integrase inhibitors, they do not affect the interpretation of the results here because the integrase inhibitors were applied continuously throughout the experiments [28, 30, 31].

Due to the expected dynamics in the presence of on-going replication, a dynamic model of 2-LTR concentration is able to quantify the amount of on-going replication [32]. However, since the IRB imposed strict limitations on the amount of blood that can be drawn from a patient during a clinical trial, only a few samples can be taken with which to fit the model [30, 32]. In this paper, we introduce a robust, systematic method of experiment design to maximize the information content under the constraints of the IRB.

## Materials and methods

### 2-LTR model

In order to analyze the data from the Buzon study, Luo et al. developed a mathematical model of 2-LTR circle production following treatment intensification with an integrase inhibitor [32, 36]. Their model is comprised of a two-state ordinary differential equation. The two states represent the concentration of 2-LTR circles and the concentration of actively infected CD4+ T-Cells in the blood. The model takes the form:

$$\begin{array}{c} \begin{array}{cc} \dot{y} & =-\left(1-\left(1-\eta_{II}u_{II}\right)R\right)ay+y_{e}\\ \dot{c} & =\phi k_{II}\left(1-\eta_{II}u_{II}\right)R)ay+k_{II}\eta_{II}u_{II}Ray-\delta c \end{array} \end{array}$$ (1)

where **y** is the concentration of actively infected CD4+ T-Cells and **c** is the concentration of 2-LTR circles. The term **y**_**e**_, is in an input into the system and represents entry of actively infected cells from exogenous sources, such as activation of latently infected cells. In both the integrase intensification study done by Buzon et al. and the similar study done by Hatano et al., the patients’ viral load had been ART-suppressed for at least six months. From this, it is safe to assume that the dynamics have reached steady state at the beginning of the experiment when the integrase inhibitor is first administered. Based on this assumption, the 2-LTR concentration in Eq (1) can be simplified as follows:

$$\begin{array}{c} \begin{array}{cc} & c\left(t\right)=c\left(\infty \right)+\left(c\left(0\right)-c\left(\infty \right)\right)e^{-\delta t}\\ & +c\left(\infty \right)\frac{\delta \eta_{II}R\left(e^{-\delta t}-e^{-a(1-\left(1-\eta_{II}\right)R)t}\right)}{\left(1-R\right)(a\left(1-\left(1-\eta_{II}\right)R\right)-\delta )} \end{array} \end{array}$$ (2)

with a steady state initial value of

$$\begin{array}{c} c\left(0\right)=\frac{k_{II}y_{e}\phi R}{\delta (1-R)} \end{array}$$ (3)

and the final value of

$$\begin{array}{c} c\left(\infty \right)=\frac{k_{II}y_{e}\left(\phi +\eta_{II}-\phi \eta_{II}\right)R}{\delta (1-\left(1-\eta_{II}\right)R)} \end{array}$$ (4)

Parameter definitions and units are defined in Table 1.

**Table 1:** Model parameter definitions.

Parameter	Definition	Units
**y**	concentration of infected cells	cells/10^6^PBMC
**c**	concentration of 2-LTR circles	2LTR/10^6^PBMC
R	probability infected cell infects a target cell in a generation	unitless
a	death rate of actively infected cells	*day*^−1^
**y**_*e*_	rate of exogenous production of infected	infected cells/ 10^6^PBMC×Day
*η*_*II*_	Ratio-reduction in R following integrase inhibitor intensification	unitless
**u**_*II*_	binary variable: 1 when integrase inhibitor is applied and 0 when it is not	unitless
*ϕ*	Ratio of probability of 2-LTR formation with integrase inhibitor vs. without	unitless
k_*II*_	The probability of 2-LTR circle formation when integrase inhibitor is present	2LTR/ infected cells
*δ*	decay rate of 2-LTR circles	day^−1^

### Measurement error

The noise in the measurement process primarily stems from two sources [37]. The first is the noise inherent in sampling a volume of blood from the body. This sample is not always an exact representation of the true blood concentration. Given a small sample size relative to the total blood volume, the number of particles in the sample follows a Poisson distribution with a probability mass function (PMF).

$$\begin{array}{c} P\left(n|v\times c\right)=\frac{\left(v\times c\right)\cdot e^{-(v\times c)}}{n!} \end{array}$$ (5)

where v is the sample volume, c is the true concentration in the blood, and n is the total number of particles present in the sample.

The second major contributor to measurement noise comes from the quantification process. The amount of noise from the quantification process is a function of which technique is used. The most common technique for quantifying DNA is real time polymerase chain reaction (qPCR). In this method the samples are amplified and then quantified using a labeling probe. This process gives rise to a qPCR assay probability mass function

$$\begin{array}{c} \begin{array}{cc} P(m|n) & =\{\begin{array}{cc} \frac{exp(-\frac{{\left(ln\;m-ln\frac{n}{v}\right)}^{2}}{2\sigma {\left(n\right)}^{2}})}{\sqrt{2\pi }m\sigma \left(n\right)}, & m>0\\ 1, & m=0\\ 0, & Otherwise \end{array} \end{array} \end{array}$$ (6)

where *σ*(*n*) = *ln*10 × 10^−0.21−0.24*log*_10_*n*^ is the equation for the log normal standard deviation of the qPCR growth process as a function of viral concentration [37].

### Traditional optimality methods

The majority of the common measures of optimality focus on maximizing the information content in a given experiment. This is usually done by optimizing some criteria of the Fisher’s information [3, 38, 39]:

$$\begin{array}{c} M\left(\Theta \right)=\int {\left(\frac{\partial }{\partial \Theta }log\;f\left(x;\Theta \right)\right)}^{2}f\left(x;\Theta \right)dx \end{array}$$ (7)

which can be expressed as a matrix

$$\begin{array}{c} M{\left(\Theta \right)}_{kl}=E[(\frac{\partial }{\partial \Theta_{k}}log\;f\left(x;\Theta \right))(\frac{\partial }{\partial \Theta_{l}}log\;f\left(x;\Theta \right))|\Theta ] \end{array}$$ (8)

where Θ is a vector containing the HIV 2-LTR model parameters.

The choice of which criteria to optimize can be highly subjective. In this paper we analyze the outcome of four frequently used design paradigms, A-optimal, D-optimal, T-optimal, and E-optimal. We then compare them to our novel Expected Kullback-Leibler method.

#### A-optimal design

The A-optimal method seeks to minimize the trace of *M*(Θ)^−1^ and in doing so minimizes the sum of the variances [3, 40].

$$\begin{array}{c} min\{\left(1/m\right)trace\left(M{\left(\Theta \right)}^{-1}\right)\}\Rightarrow T \end{array}$$ (9)

where *T* is a vector of n sample points in the study *T* = [*t*_1_, *t*_2_, …, *t*_*n*_]. This optimization paradigm is commonly chosen due to its relative mathematical simplicity. In the case of simple linear models, closed form A-optimal solutions can often be found.

#### D-optimal design

The D-optimal method is perhaps the most commonly used optimality criteria and seeks to minimize determinant of the information.

$$\begin{array}{c} max\{det\left(M{\left(\Theta \right)}^{-1}\right)\}\Rightarrow T \end{array}$$ (10)

In doing so it minimizes the m-dimensional ellipsoid of the maximum region of confidence for the maximum likelihood estimate of the parameters Θ [3, 40]. This in essence minimizes the covariance of the parameter estimates.

#### T-optimal design

The T-optimal method is another criterion often used due to its relative mathematical simplicity. This method simply seeks to maximize the trace of the information matrix *M*(Θ).

$$\begin{array}{c} max\{trace(M(\Theta ))\}\Rightarrow T \end{array}$$ (11)

While this method is straightforward mathematically and computationally simple, it often leads to unsatisfactory results [3, 40].

#### E-optimal design

The fourth and final traditional method of optimization that is considered in this paper is the E-optimal design approach. Similar to the D-optimal design, the E-optimal technique is a bit more computationally intensive. It seeks to maximize the minimum eigenvalue of the information matrix *M*(Θ).

$$\begin{array}{c} max\lambda_{min}\left\{\left(M\left(\Theta \right)\right)\right\}\Rightarrow T \end{array}$$ (12)

This method is often used as an alternative to the D-optimal method when one more of the parameters has a relatively large variance in comparison to the other parameters. Graphically, the E-optimal method minimizes the maximum diameter of the m dimensional ellipsoid [3, 40].

There are numerous other traditional optimality criteria; however, these four were chosen to demonstrate how the various methods result in different optimal designs and to compare common methods to our novel approach.

### Expected Kullback-Leibler Divergence (EKLD)

#### Simulated patient pool

To evaluate a sample schedule, we must evaluate it against a number of possible patients. The posterior distributions developed by the Luo et al. analysis of the Buzon data represents the range of possible parameter values possible patients may have [32, 36].

The multivariate distribution is constructed from a set of five system parameters Θ(*A*, *ϕ*, *R*, *η*_*II*_, *δ*). Parameters R, *ϕ*, *η*_*II*_, and *δ* are exactly established from Eq 1. Parameter A was derived as an observable parameter which reduces the covariance between other parameters [32, 36].

$$\begin{array}{c} A\equiv \frac{k_{II}y_{e}R}{\delta } \end{array}$$ (13)

In order to evaluate a sampling schedule’s performance across multidimensional multivariate distribution while maintaining computational tractability, we apply an unscented transform to obtain 2*N* + 1 simulated sigma point patients which maintain the same first and second moment characteristics as the initial multivariate distribution [41].

$$\begin{array}{c} \begin{array}{cc} X_{i} & =\{\begin{array}{cc} \mu , & i=0\\ \mu +\sqrt{N\Sigma_{i}}, & 1\leq i\leq N\\ \mu -\sqrt{N\Sigma_{i}}, & N<i\leq 2N \end{array}\\ X_{i} & =(A_{i},\phi_{i},R_{i},\eta_{IIi},\delta_{i}) \end{array} \end{array}$$ (14)

where each *X*_*i*_ is the separate set of parameters for each sigma point patient. The *μ* and Σ_*i*_ terms are the mean of the prior distribution and the *i*_*th*_ column of the covariance matrix of the prior distribution respectively. N is the total number of dimensions in our prior distribution, which in this case is a five-dimensional distribution.

**Fig 1:**
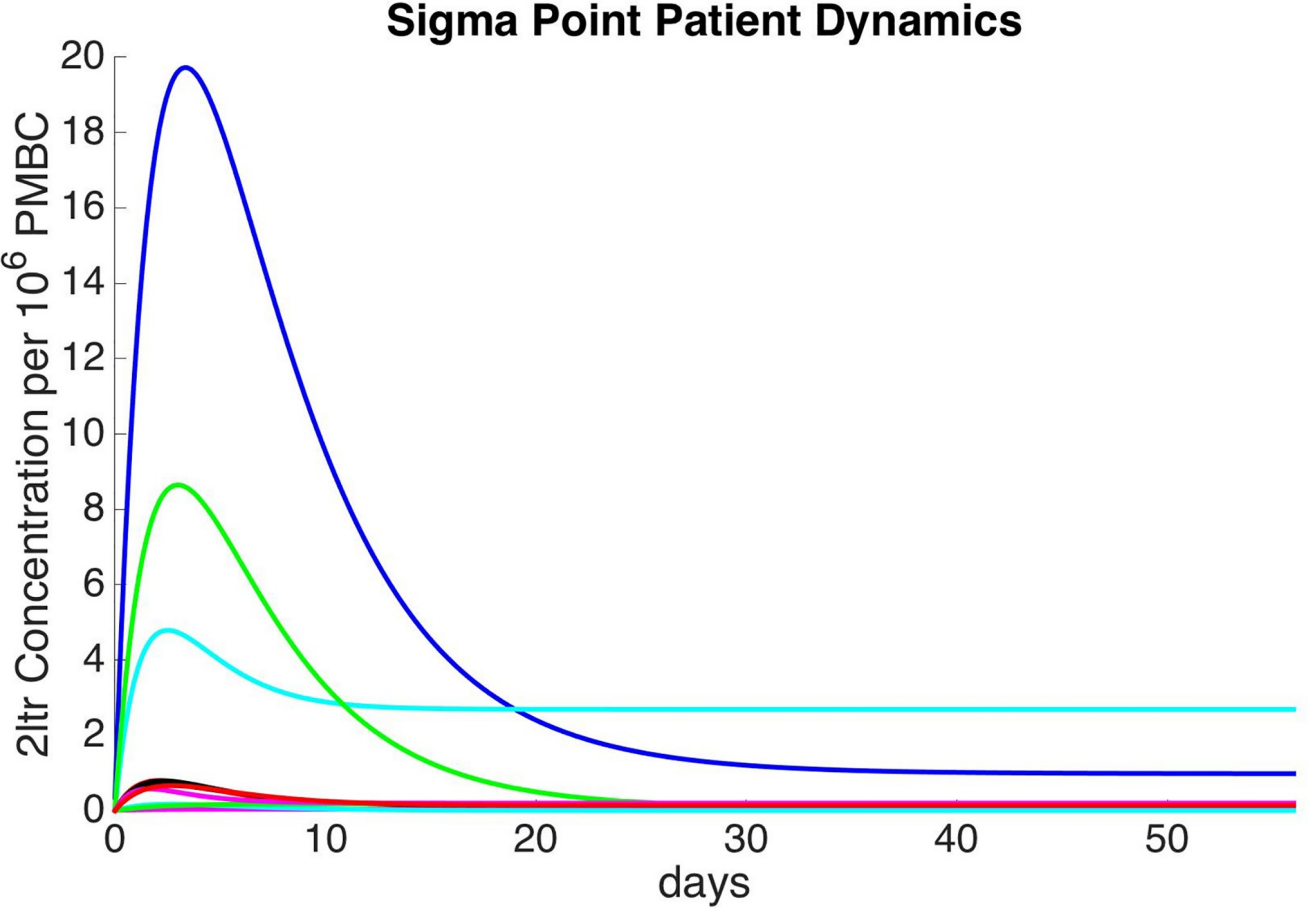
Sigma point patient dynamics.

The five-dimensional parameter space results in 11 sigma point patients that exhibit a range of 2-LTR concentration dynamics as illustrated in Fig 1. The transient nature of the dynamics is evident from the various trajectories.

#### Markov Chain Monte Carlo (MCMC) Methodology

For each candidate schedule we construct simulated data based on our models and measurement noise. The posterior distributions for parameter set Θ_*i*_ are constructed for each patient *i* using a Markov Chain Monte Carlo technique. We define **c**(*t*_*k*_, Θ_*i*_) as the true concentration measured at sample point k *i* using parameter set Θ_*i*_.

With a qPCR assay we assume measurement noise consistent with the assay which leads to measurements as

$$\begin{array}{c} m_{ik}=lnN\left(ln\left(c\left(t_{k},\Theta \right)\right),\ln\left(10\right)\sigma \left(n\right)\right) \end{array}$$ (15)

Applying Bayes theorem, we arrive at the equation

$$\begin{array}{c} P\left(\Theta_{i}|m_{ik}\right)=\frac{L\left(\Theta_{i}|mik\right)P\left(\Theta_{i}\right)}{\int_{0}^{\infty }P\left(m_{ik}|\Theta_{i}\right)P\left(\Theta \right)d\Theta } \end{array}$$ (16)

However, $\int_{0}^{\infty }P\left(m_{ik}|\Theta_{i}\right)P\left(\Theta \right)d\Theta$ is a constant scaling factor of the posterior distribution [36, 37]. For computational simplicity we simplify and arrive at the equation

$$\begin{array}{c} P\left(\Theta_{i}|m_{ik}\right)\propto L\left(\Theta_{i}|m_{ik}\right)P\left(\Theta_{i}\right) \end{array}$$ (17)

which has the same form and conserves the KLD [33].

#### KLD calculation

Calculation of the Kullback Leibler Divergence (KLD) between the five-dimensional multivariate prior and posterior distributions is done using equation

$$\begin{array}{c} \begin{array}{c} KLD\left(T\right)=\frac{1}{2}(\log_{2}(\frac{det\;\Sigma_{2}}{det\;\Sigma_{1}})-n-tr\left(\Sigma_{2}^{-1}\Sigma_{1}\right)+{\left(\mu_{2}-\mu_{1}\right)}^{T}\Sigma_{2}^{-1}\left(\mu_{2}-\mu_{1}\right)) \end{array} \end{array}$$ (18)

where (*μ*_1_, Σ_1_) and (*μ*2, Σ_2_) are the mean vector and covariances matrices of the prior and posterior multivariate distributions respectively and *n* is the number of dimensions in the distribution [33, 38]. This formula for KLD can be used when both distributions are normal, or, as in our case, are normalized through transformation. Because the base-2 logarithm is used in the calculation of the KLD, the result is measured in base-2 units of information (bits).

We should also note that Eq 18 is applicable when all of the parameters are normally distributed. log(*A*),log(*ϕ*),and log(*δ*) are normally distributed. Parameters *η*_*II*_ and *R* are transformed using the normal distribution quantile function. The KLD between distributions is conserved through all transformations [33, 38].

The Expected Kullback Leibler Divergence (EKLD) is estimated by calculating the KLD for each sigma point patient and multiplying by the probability of the patient occurring based on the parameter distributions calculated by Luo et al.

$$\begin{array}{c} EKLD\left(T\right)=\sum_{i=1}^{11}KLD{\left(T\right)}_{i}P\left(\Theta_{i}\right) \end{array}$$ (19)

Where i represents a patient from the simulated sigma point patient pool sampled from the Prior Distribution. Calculating the KLD only the 11 sigma point patients is a simplification made to make the calculation more computationally feasible. The true EKLD would require the KLD is calculated and integrated over the entire prior distribution.

$$\begin{array}{c} EKLD\left(T\right)=\int_{i}KLD{\left(T\right)}_{i}P\left(\Theta_{i}\right) \end{array}$$ (20)

It has been shown the sample schedule order is preserved when KLD is calculated using the simplified method in lieu of integrating over the entire distribution [42].

### Genetic algorithm

The inherent binary nature of time series measurements, taking a sample on a given day or not in this case, lends itself well to a genetic algorithm optimization method. For such a system, it is reasonable to assume that closeness of two candidate schedules on a Hamming distance measure will correlate with closeness on a measure of information gained. To construct the GA, candidate sample schedules are represented by a chromosome. Each chromosome consists of genes and each gene is further broken down into base pairs. Each base pair represents a potential sample day and takes on a binary value, 0 for days at which no sample and 1 for days at which a sample is taken. The genes combine to form a chromosome with total base pairs equal to the total number of possible sample days.

The algorithm is run with 20 child chromosomes per generation. The first generation is created by randomly selecting the appropriate number of base pairs (sample days) per chromosome. The corresponding information content is then calculated for each chromosome by calculating its associated KLD. The chromosomes are then ranked in terms of the relative fitness by assigning chromosomes yielding higher KLD values a greater fitness level. Chromosomes with the highest fitness are then used as the parent solutions to create the children for the next generation. Children are created through a process of genetic crossovers and mutations [43–45].

Genes are able to crossover to different locations or to the same location between parents. Point mutations occur after the crossovers to ensure that the chromosome has exactly number of sample points desired. These mutations occur by bit inversion. This is to ensure that the algorithm is able to escape local minima.

## Results and discussion

### Six-point optimal sampling schedule

In the previous integrase inhibitor intensification HIV 2-LTR study done by Buzon et al., a total of six samples were taken per patient. The samples were taken at day 0, day 14, day 28, day 84, day 168, and day 336. We chose to find the optimal six-sample schedule, for all of the optimality criteria, to compare to the schedule used in the Buzon experiment.

**Fig 2:**
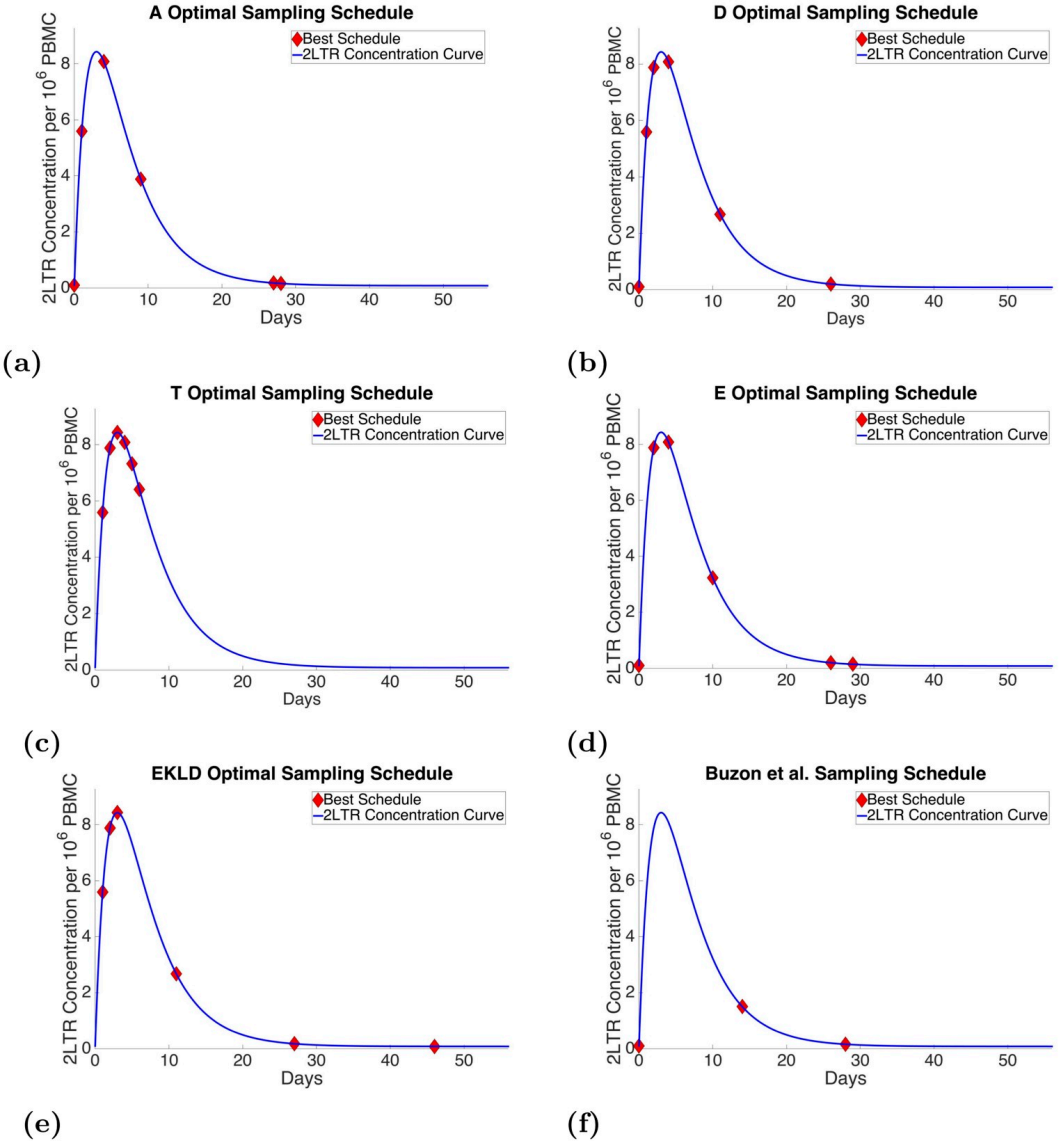
6 point sample schedules.

As can be seen in Fig 2a through Fig 2e the best six-point sample schedules for each optimality criterion are plotted with the 2-LTR concentration curve plotted over an 8-week time frame. The red diamond markers represent when a sample would be taken under each respective optimality criterion.

The A-optimality criterion, which seeks to minimizes the sum of the variances of the parameters, yielded samples at day 0, day 1, day 4, day 9, day 27, and day 28. A sample at day 0 will quantify the initial condition. The sample at day 1 as the concentration is increasing will allow for information about the rate of 2-LTR production. Next the criterion seeks a point near the peak and then a point during the decay phase of the 2LTR. the last two points are close together at the bottom of the curve.

The D-optimality criterion, which seeks to minimize the region of confidence of the maximum likelihood estimate of the parameters, resulted in a schedule similar to that of the A-optimality criterion. However instead of choosing two points at the bottom of the curve, after the decay of the 2-LTR concentration, it chose two points close together during the initial rise of the 2-LTR concentration. The D-optimality criterion chose samples at day 0, day 1, day 2, day 4, day 11, and day 26.

The T-optimality criterion, which seeks to minimize the trace of the information matrix, returned a schedule with heavy contrast to the other schedules. This criterion yielded samples points collected around the peak of the 2-LTR curve with consecutive samples at day 1, day 2, day 3, day 4, day 5, and day 6.

The E-optimality criterion, which seeks to minimize the maximum diameter of the m dimensional parameter estimate ellipsoid, yielded a sample schedule very similar to that of the A-optimality criterion, however, this criterion preferred a sample point closer to the peak instead of during the ascent of the 2-LTR concentration as in the A-optimality schedule. The E-optimality criterion chose samples at day 0, day 2, day 4, day 10, day 26 and day 27.

Our method, the EKLD, unlike the A, D, and E optimality criterion did not elect to take a sample at the day 0. Instead the optimal EKLD schedule chose three consecutive points during the rise of the 2-LTR concentration up to the peak. The samples are then spread out with 1 on the descent of the concentration, and two at the bottom spaced out by several days. The optimal EKLD sample schedule chose points at day 1, day 2, day 3, day 11, day 27 and day 46.

Buzon et al. in contrast to all of the optimal schedules took six samples that are much more spread out at day 0, day 14, day 28, day 84, day 168, and day 336. As both the preliminary data and our model suggests, the majority of the dynamics in the 2-LTR concentration occur within the first four weeks post-intensification. Only three samples from the schedule used by Buzon et al., were taken during this period. The other three were taken much later and in fact were not taken within the 8-week window shown in Fig 2f.

**Fig 3:**
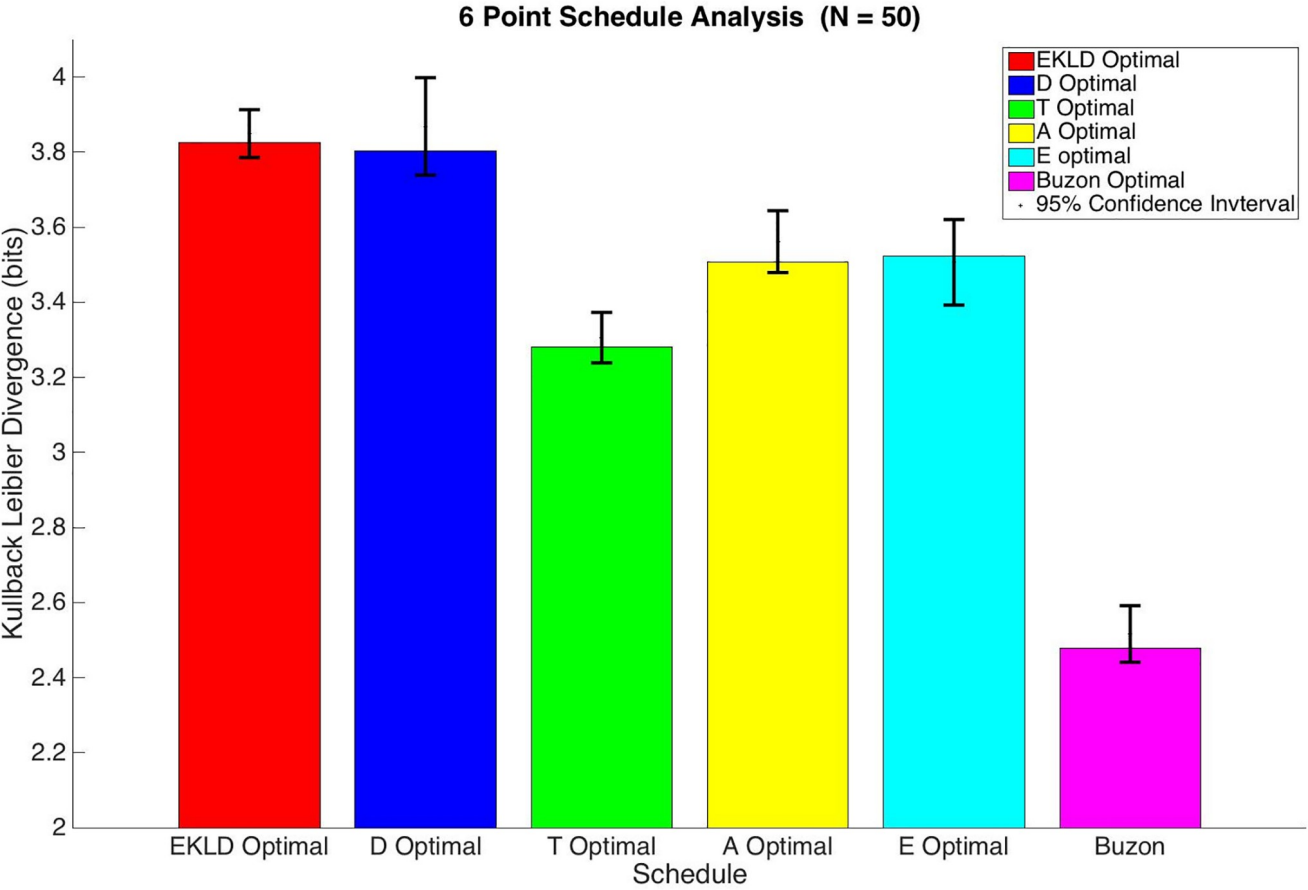
6 point schedule analysis.

In order to compare the performance of each schedule, we calculate the Expected Kullback-Leibler Divergence for all of them. Because the EKLD is a Bayesian based global method of estimating the divergence of the posterior parameter distribution from the prior distribution it can serve as a fair measure for comparison. The results of this analysis are shown in Fig 3.

Based on the results shown in both Table 2 and Fig 3 the both Expected Kullback Leibler Divergence based schedule and the D-optimality base schedule yielded significantly more information than any of the other schedules with a median gain in information of around 3.8 bits for each. The A-optimality and E-optimality based schedules also produced a similar gain in information of around 3.5 bits for each. The T-optimality based schedule performed significantly worse than all of the other optimal schedules with a median gain information of 3.28 bits. As expected all of the optimal schedules vastly outperformed the schedule used by Buzon et al. Their sample schedule only produced a gain in information of 2.48 bits. Because KLD is measured on a log scale (bits), this means the EKLD and D-optimal schedules would produce over 2.5 times more information than the schedule used in the experiments done by Buzon et al., without any increase in total number of measurements or study cost.

**Table 2:** Six-point sample schedules.

	Samples						EKLD
	1	2	3	4	5	6	
A-optimal	0	1	4	9	27	28	3.51
D-optimal	0	1	2	4	11	26	3.80
T-optimal	1	2	3	4	5	6	3.28
E-optimal	0	2	4	10	26	27	3.52
EKLD	1	2	3	11	27	46	3.83
Buzon	0	14	28	84	168	336	2.48

### Four-point optimal sampling schedule

In a follow-up study to the Buzon experiment, another integrase inhibitor intensification experiment was carried out by Hatano et al., however with less samples per patient. In their trial Hatano et al., took patient samples earlier in the experiment, however only four samples were taken. The samples were taken at Day 0, Day 7, Day 14 and Day 56. As a comparison to the Hatano sample schedule we found the optimal four-point sample schedules for the various optimality criteria.

**Fig 4:**
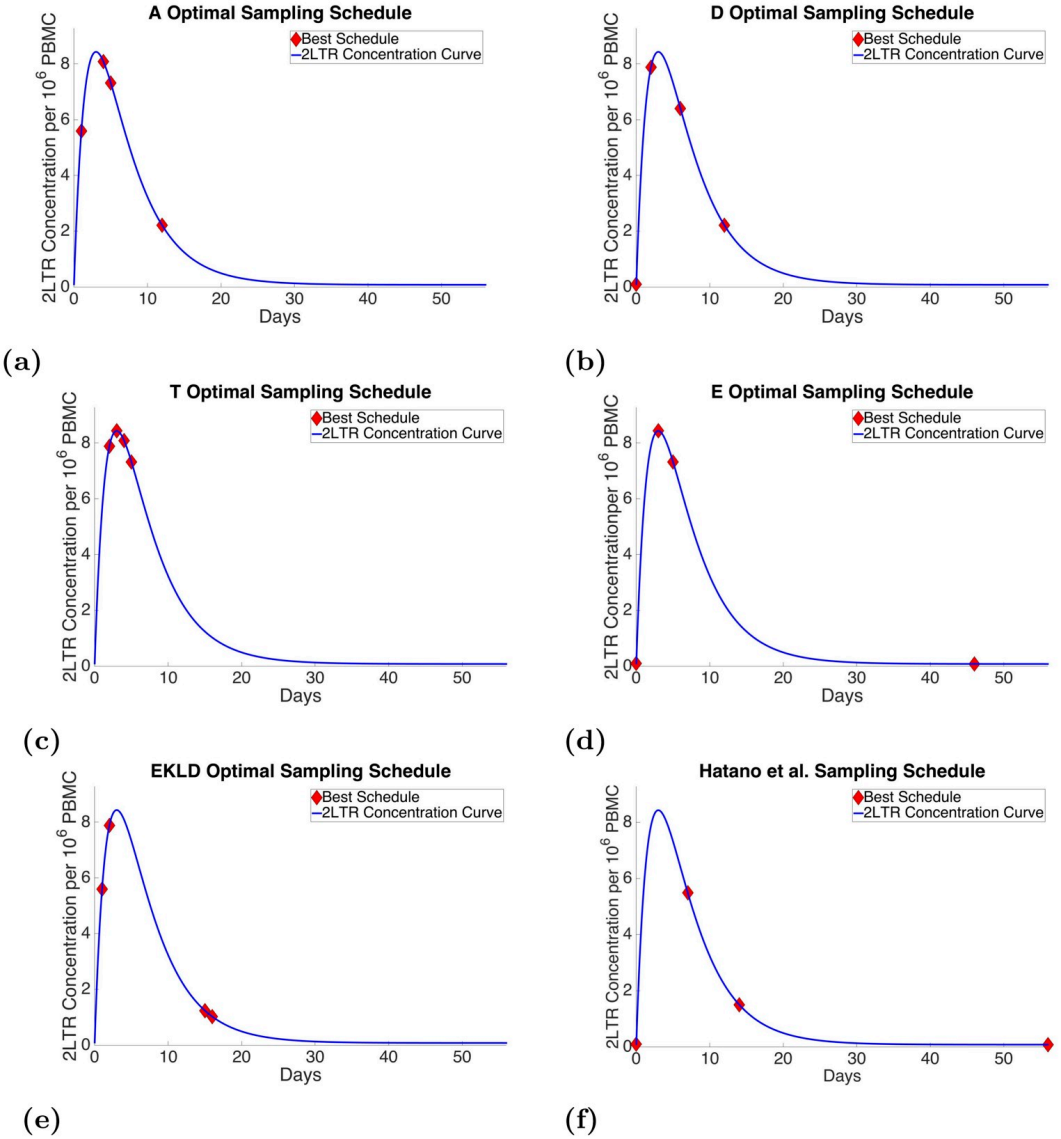
4 point sample schedules.

Fig 4a through Fig 4e show the best four-point schedules for all of the optimality criteria plotted with the 2-LTR concentration curve over an 8-week time frame with the sample points denoted by the red diamond marker.

The best four-point schedule under the A-optimality criterion is shown in Fig 4a with samples taken at day 1, day 4, day 5 and day 12. In the six-sample schedule for the A-optimality criterion a sample was taken at day 0; however, this sample was not present in the four-sample optimal solution for this criterion. The six-sample optimal also chose two points toward the bottom of the 2-LTR concentration curve. The four-sample optimal, on the other hand, contained more points around the peak of the curve and one capturing the decay of the 2-LTR concentration on the downslope.

For the D-optimality criterion, the best four-point schedule is shown in Fig 4b with samples taken at day 0, day 2, day 6, and day 12. This criterion kept the sample at day 0 that was present in the six-sample optimal; however, similar to the A-optimality criterion schedule, the four-sample schedule did not include the points that were selected further out in the schedule. This criterion instead focuses on the initial concentration, a point on near the peak, and two points on the descending side.

The best four-point schedule under the T-optimality criterion is shown in Fig 4c with samples taken at day 2, day 3, day 4, and day5. This sample schedule is very similar to the six-sample optimal for this criterion. All of the samples are taken consecutively around the peak. The difference between the six-sample optimal and the four-sample optimal are that first and last points of the six-sample optimal, taken at day 1 and day 6 respectively, are removed for the four-sample solution.

For the E-optimality criterion, the best four-point schedule is shown in Fig 4d with sample points taken at day 0, day 3, day 5 and day 46. This criterion also keeps the sample at day 0 that was present in the six-sample optimal. Unlike the schedules selected by the other optimality criterion, the E-optimality criterion chose a point that was relatively late in the experiment at day 46. Because the E-optimality criterion focuses on minimizing the maximum diameter of the ellipsoid this suggest that there exists some information about at least one of the parameters at later time points in the experiment, which might have a relatively large variance when compared to the other parameters.

The best four-point schedule using the EKLD method is shown in Fig 4e with samples at day 1, day 2, day 15 and day 16. The EKLD method also similar to the A and D criterion did not choose point later in the experiment four the four-point optimal, where it had selected points relatively late in the experiment in the six-sample optimal at day 27 and day 46. Instead the EKLD optimal schedule has two points consecutive points on each side of the curve, with the points on the descending side of the curve toward the bottom, where the dynamics display a high degree of curvature.

The schedule used by Hatano et al. in their experiment is shown in Fig 4f. In their experiment samples were taken at day 0, day 7, day 14 and day 56. Similar to the E-optimal solution, the sample schedule used by Hatano et al. takes samples at the day 0 and a sample later in the experiment. One key difference however, is that the remaining two samples are taken near the peak of the 2-LTR concentration dynamics in E-optimality solution, whereas they are taken after the peak in the Hatano et al. experiment.

**Fig 5:**
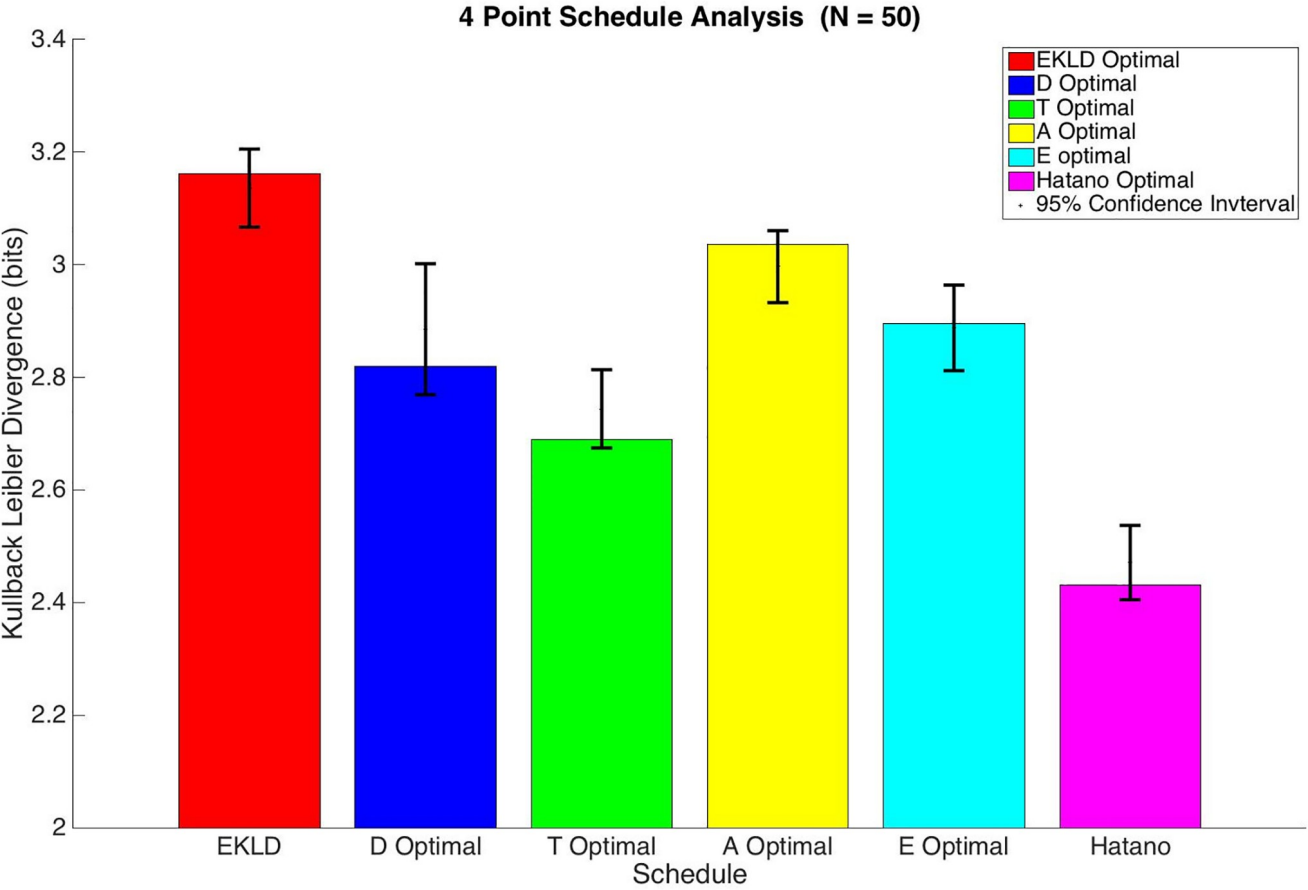
4 point schedule analysis.

In order to compare the schedules against one another we calculated the EKLD for all of them as a fair measure of information gain. The results of this analysis are illustrated in the bar graph in Fig 5.

Based on the results shown in Table 3 and Fig 5, the EKLD schedule yielded a significantly greater gain in information than any of the other schedules with an information gain of 3.16 bits. This is in contrast to the analysis done for the six-sample optimal where the D-optimality criterion produced a schedule that produced a similar gain in information as that of the EKLD schedule.

**Table 3:** Four-point sample schedules.

	Samples				EKLD
	1	2	3	4	
A-optimal	1	4	5	12	3.04
D-optimal	0	2	6	12	2.82
T-optimal	2	3	4	5	2.69
E-optimal	0	3	5	46	2.90
EKLD	1	2	15	16	3.16
Hatano	0	7	14	56	2.43

Out of all of the schedules from the traditional optimality methods, the schedule from D-optimality criterion produced the highest gain in information for the six-sample method; however, with four samples the A-optimality method yielded the highest gain in information with 3.04 bits. The E-optimality schedule produced the next highest median gain in information with 2.9 bits but was not significantly different than the expected gain in information from the D-optimality which had a median gain in information of 2.82 bits. The T-optimality criterion again produced the worse performing of all of the optimal solutions with a median information gain of 2.69 bits.

Even though there is a significant difference in the expected information gain for the various optimal schedules they all once again performed better than the ad hoc schedule that was used in the experiment done by Hatano et al., which yielded a gain in information of only 2.43 bits. This shows that there is value in performing the optimization prior to carrying out the experiment. The EKLD schedule will on average yield about 1.65 times more information than the Hatano et al. schedule using the same amount of data points.

### Measurement uncertainty and information content

**Fig 6:**
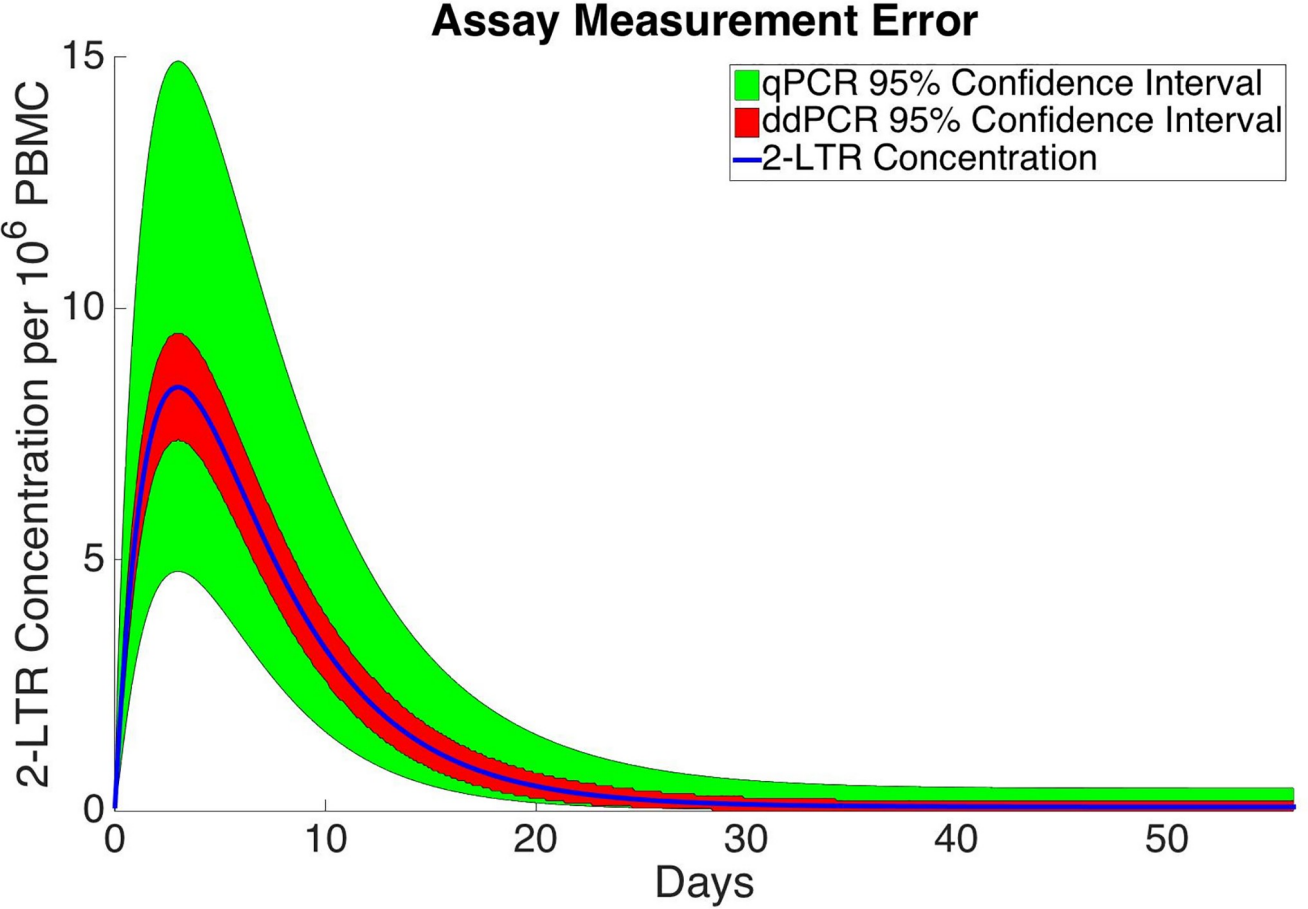
qPCR vs. ddPCR measurement error.

In the previous analysis we have assumed that measurements will be taken and quantified using a qPCR assay, the most commonly used assay in the quantification HIV DNA. There is however, another more accurate assay which is gaining popularity among the biomedical community. This new technique is known as a droplet digital polymerase chain reaction (ddPCR) assay [46, 47]. It uses microfluidics to perform PCR amplification on thousands of tiny droplets. The droplets are run through a machine and either test positive for the presence of DNA or negative. Using Poisson statistics this assay is accurately estimate the DNA concentration, especially at low copy numbers where the qPCR method fails. The primary source of noise in this method is not in the assay but in taking the initial blood sample. The difference in measurement accuracy between the qPCR assay and ddPCR assay is illustrated in Fig 6. The 95 percent prediction interval for each method is shown for the nominal patient 2-LTR concentration curve with the qPCR error shown in green and the ddPCR error shown in red.

For measurement noise consistent with a ddPCR quantification assay, measurement noise is defined as

$$\begin{array}{c} \begin{array}{cc} & m_{ik}\left(t_{k}\right)=Poiss\left(\lambda_{i}\right),\\ & \lambda_{i}=N\left(1-e^{\frac{-c(t_{k},\Theta_{i})\times v}{N}}\right) \end{array} \end{array}$$ (21)

where *N* is the number of droplets, *c* is the true concentration and *v* is the sample volume.

Using this simulated data, we then use an MCMC technique to find posterior distributions for each parameter set Θ_*i*_. The posterior distributions from previous analyses were the basis for the uninformative prior distributions *P*(Θ_*i*_). The likelihood function for our MCMC calculation takes the form

$$\begin{array}{c} L\left(\Theta_{i}|m_{ik}\right)=f_{LN}(m_{ik}\left(t_{k}\right),N\left(1-e^{\frac{-c(t_{k},\Theta_{i})\times v}{N}}\right)) \end{array}$$ (22)

where $f_{LN}$ denotes the probability mass function of the Poisson distribution function [33, 46].

**Fig 7:**
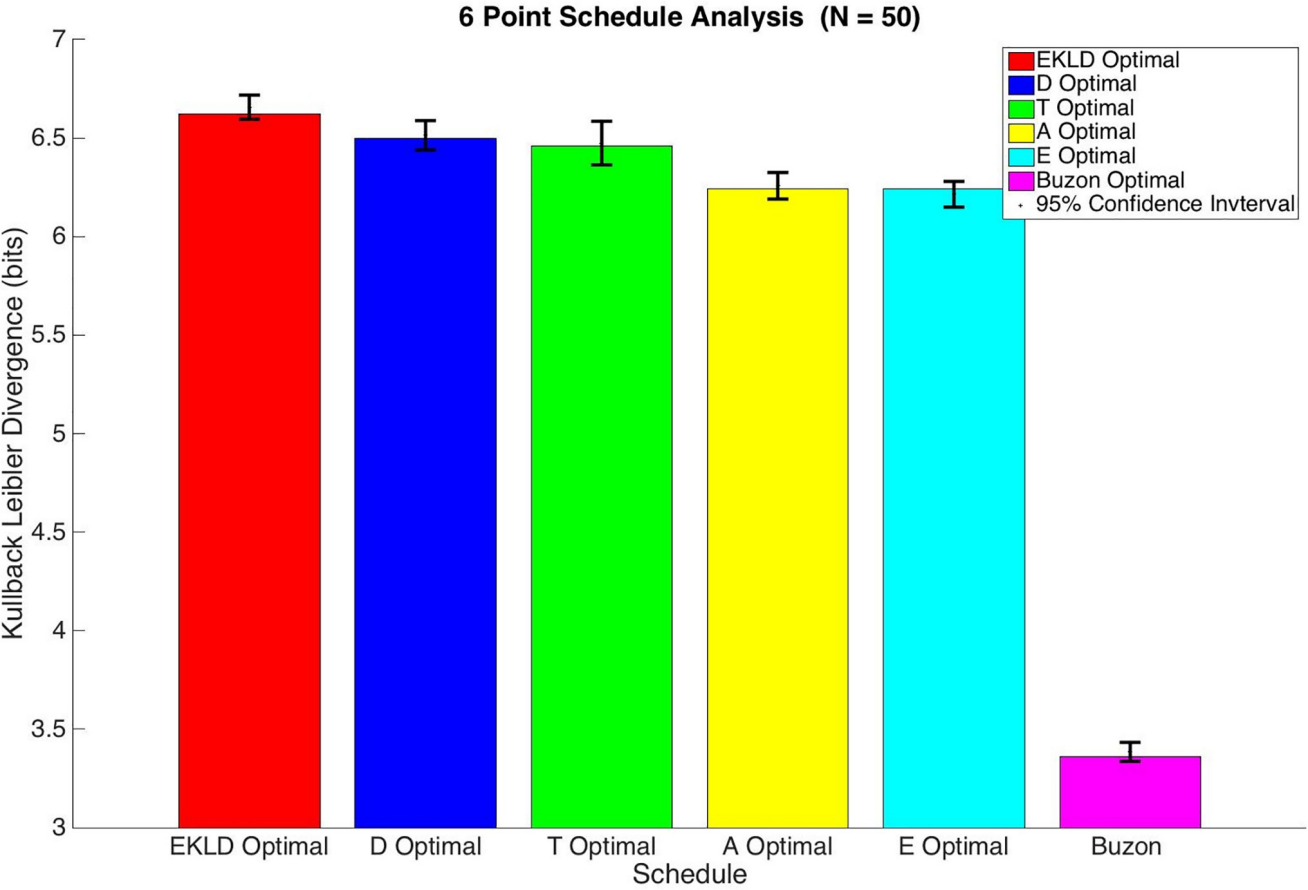
6 point ddPCR schedule analysis.

We can then calculate the EKLD under the assumption of measurement uncertainty consistent with a ddPCR assay. Fig 7 illustrates EKLD of the six-point sample schedules with ddPCR measurement noise. We still find that EKLD based schedule provided the highest median expected gain in information with 6.62 bits. This equates about seven times more information about the parameters when using a ddPCR assay versus using a qPCR assay for this schedule.

The D-optimality criterion-based schedule is the still next best performing schedule with a median expected gain in information of 6.5 bits which provides 6.47 times more information that using a qPCR assay for this schedule. The T-optimal based criterion, which yielded the smallest gain information among all of the optimal schedules for the six-point analysis with a qPCR assay, now provides a greater gain in information than the A and E optimality criterion-based schedules. The T optimality criterion-based schedule provides a median expected gain in information of 6.46 bits with the ddPCR assay. For this schedule the ddPCR assay provides approximately nine times more information than the qPCR assay.

The A and E Optimality criterion which provided a similar gain in information with a qPCR assay still perform similarly with the ddPCR assay; however, for both the expected median information gain has increased to 6.24 bits. This results in 6.66 and 6.58 times more information when using the ddPCR assay versus using the qPCR assay for the A optimality criterion schedule and E Optimality criterion schedule respectively. The Schedule used by Buzon et al. remains the worst schedule of them all in terms of expected information gain. This schedule results in a median expected information gain of 3.36 bits with a ddPCR assay which is about 1.84 times more information than would have been provided if a qPCR assay was used. The assay specific information content is shown for each six-point schedule in Table 4.

**Table 4:** Six-point sample schedules EKLD.

	Schedules					
	EKLD	D	T	A	E	Buzon
qPCR	3.83	3.80	3.28	3.51	3.52	2.48
ddPCR	6.62	6.50	6.46	6.24	6.24	3.46

**Fig 8:**
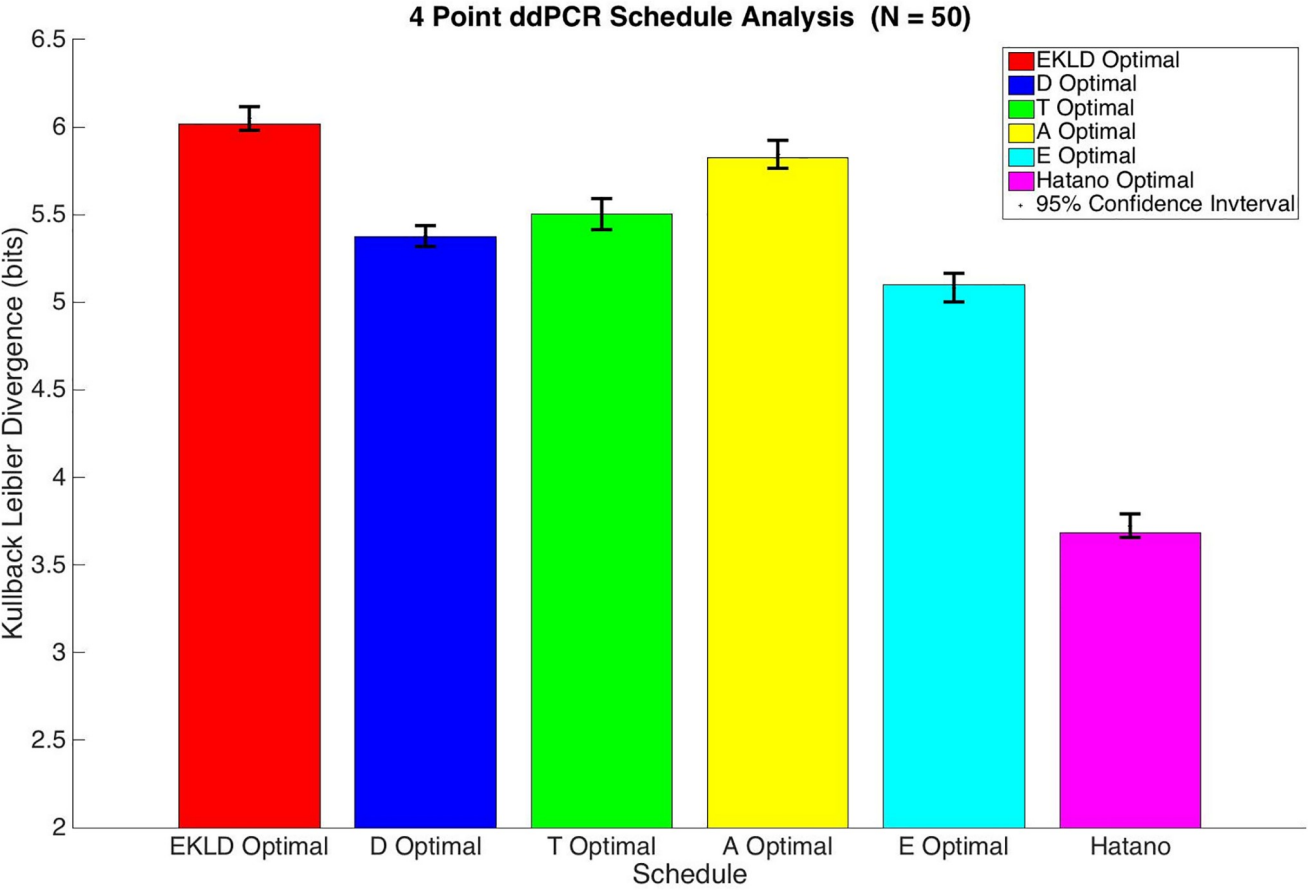
4 point ddPCR schedule analysis.

Fig 8 illustrates the EKLD of the four-point sample schedules with ddPCR measurement noise. Again, we see that there is a sizable increase in the amount of information gained for each schedule. The EKLD based schedule still has the highest median expected gain in information with a median expected information gain of 6.02 bits. When compared to the amount of information gain expected from the qPCR assay for this schedule, the ddPCR assay will provide approximately 7.24 times more information about the parameters. The A-optimality criterion schedule, as in the qPCR analysis, still has the second highest median expected gain in formation when using the ddPCR assay with a median expected gain in information of 5.83 bits. This schedule provides 6.91 times more information when using the ddPCR assay versus using the qPCR assay.

The other three schedules, on the other hand, perform slightly differently assuming ddPCR measurement noise. Now the T-optimality criterion schedule, which was the worst performing of the optimal schedules, out performs the D and E optimality criterion schedules with an expected gain in information of 5.5 bits. There will be approximately seven times more information gained in the experiment when using the ddPCR assay vs. the qPCR assay for this schedule. The D optimal criterion-based schedule yields an expected information gain of 5.37 bits, which results in 5.87 times more information using the ddPCR assay vs. the qPCR assay. The E optimal schedule is now the worst performing of the optimal schedules providing a median expected information gain of 5.1 bits. Using the ddPCR assay will provide about 4.6 times more information about the parameters vs. using the qPCR assay. The schedule used by Hatano et al. remains the worst performing schedule overall with a median expected information gain of 3.68 bits with a ddPCR assay. About 2.38 times more information will be provided if the experiment uses a ddPCR assay instead of a qPCR assay. The assay specific information content is shown for each four-point schedule in Table 5.

**Table 5:** Four-point sample schedules EKLD.

	Schedules					
	EKLD	D	T	A	E	Hatano
qPCR	3.16	2.82	2.69	3.04	2.90	2.43
ddPCR	6.02	5.37	5.50	5.83	5.10	3.68

## Conclusion

The intent of this paper is to introduce our novel Expected Kullback-Leibler Divergence (EKLD) method for optimal experiment design. We demonstrate its utility by using it to optimize HIV 2-LTR experiments and compare the results to optimal designs using traditional methods. Our results demonstrate that all of the optimization methods provided a greater expected gain in information than the ad hoc schedules used in the two previous studies done by Hatano et al. and Buzon et al. The EKLD based schedules consistently outperformed the other optimal schedules; however, it did not always provide a significant gain in information over other optimal schedules. Even so, due to the Bayesian nature of our analysis and the inclusion of the a priori parameter information, we believe it provides a more exhaustive analysis of optimal experiment design [3, 5].

HIV 2-TLR trials can serve as a critical procedure to use to determine if and to what extent ongoing viral replication is occurring within treated HIV(+) patients. This knowledge is essential as it will guide the individualized patient treatment of the disease. In order for the procedure to useful, it must garner the maximum amount of information about the patients’ replication as possible [2]. This drives the need to optimize the trial with analyses such as those presented in the paper. The results of our analysis show that sample times, number of samples, and sample assay all have a large effect on the amount of information that the experiment will provide.

In general, monetary cost and patient burden are two of the main concerns when conducting any clinical trial. Our method can easily be extended to other clinical trials. A recent review paper of a study of clinical trials stated that each additional month for phase III clinical trials translates into a median $671,000 spent [1, 2]. In our analysis we showed that the optimal four-point EKLD based sample schedule provided approximately 1.6 times more information than a schedule used by Buzon et al. and only took 4 samples over a 16-day period, whereas the Buzon et al. experiment spanned 336 days and took 6 samples. A great deal more information was provided with fewer samples and in less time. This would translate to a great deal of cost savings for the experiment and a sizable reduction in overall burden to the patient.
